# On-Board Monitoring of SO_2_ Ship Emissions Using Resonant Photoacoustic Gas Detection in the UV Range

**DOI:** 10.3390/s21134468

**Published:** 2021-06-29

**Authors:** Mahmoud El-Safoury, Miguel Dufner, Christian Weber, Katrin Schmitt, Hans-Fridtjof Pernau, Bert Willing, Jürgen Wöllenstein

**Affiliations:** 1 Fraunhofer Institute for Physical Measurement Techniques IPM, 79110 Freiburg, Germany; mahmoud.el-safoury@ipm.fraunhofer.de (M.E.-S.); Miguel.dufner@ipm.fraunhofer.de (M.D.); christian.weber@ipm.fraunhofer.de (C.W.); katrin.schmitt@imtek.uni-freiburg.de (K.S.); juergen.woellenstein@ipm.fraunhofer.de (J.W.); 2 Department of Microsystems Engineering—IMTEK, University of Freiburg, 79110 Freiburg, Germany; 3 Rüeger SA, Ch. de Mongevon 9, 1023 Crissier, Switzerland; bw@rueger.com

**Keywords:** resonant photoacoustic detection, combustion gas monitoring, sulphur dioxide (SO_2_), container ship emissions, UV LED, MEMS microphone

## Abstract

A photoacoustic gas detector for SO_2_ was developed for ship exhaust gas emission monitoring. The basic measurement setup is based on the absorption of electromagnetic radiation of SO_2_ at 285 nm wavelength. A commercially available ultraviolet (UV) light-emitting diode (LED) is used as the light source and a micro-electro-mechanical system (MEMS) microphone as the detector. In order to achieve the required detection limits in marine applications, a measuring cell which allows an acoustically resonant amplification of the photoacoustic signal was developed and characterized. A limit of detection of 1 ppm was achieved in lab conditions during continuous gas flow. Long-term measurements on a container ship demonstrated the application relevance of the developed system.

## 1. Introduction

Ship air pollution harms health by causing respiratory diseases, and causes overall air quality problems, which have a negative impact on the natural environment (e.g., acid rain) [1]. The Annex VI of the International Convention for the Prevention of Pollution from Ships (MARPOL) sets limits on sulphur oxide (SO_X_) and nitrogen oxide emissions from ship exhausts [1]. It has limited the SO_X_ emission in specified emission control areas (ECAs) to 0.10% since 1 January 2015 [1]. The revised MARPOL Annex VI overthrew the initially set 3.50% as the global sulphur limit (outside of the ECAs) and reduced it to 0.50%, effective since 1 January 2020 [2]. In order to assure the implementation of these strict regulations, a direct measurement and monitoring method has to be applied [2]. The emitted gases should be continuously measured and analyzed, which requires the measuring device to be installed to the ship funnel. If the container ships use a cheap heavy fuel oil with high sulphur content, an exhaust gas cleaning system (EGCS) with a monitoring device for the cleaned gas must be integrated into the funnel. An EGCS partially removes the harmful components (like SO_X_ and nitrogen oxides) from the exhaust gases. On the other hand, container ships that use expensive fuel with a low sulphur content also need to verify the obedience to the imposed strict regulations, which is given through an additional monitoring device. A few years ago, measuring systems with electrochemical gas sensors were offered for this measurement challenge, but they proved to be unsuitable for the real application. These systems are no longer on the market. To the best of our knowledge, there are currently two optical gas sensor systems on the market. The MARSIC300 system from SICK, which is based on classic non-dispersive ultraviolet spectroscopy (NDUV) technology and uses a broadband ultraviolet (UV) lamp as the light source [3]. SICK devices are in the meantime installed on ships. Another supplier is the company Gasera from Finland. Gasera offers a non-resonant photoacoustic multi-gas analyzer (GaseraOne) that is claimed to be suitable for monitoring ship emissions [4]. We are not sure if the analyzers have already been installed on ships. There is also no further information on their website. However, an affordable ship exhaust gas emission monitoring system with a reliable sensitivity of 1 part per million (ppm) operating in harsh environments is still not commercially available.

Photoacoustic (PA) gas detection has proven to be a stable and precise detection method, which has grown in popularity during the last few decades. A photoacoustic detector basically requires a light source, a photoacoustic cell and an acoustic transducer [5]. The light sources most used are lasers, light-emitting diodes (LEDs) and thermal broadband infrared sources [6]. Cantilevers, quartz tuning forks or microphones are the three most common acoustic transducers to convert the photoacoustic signal into an electrical signal [6]. Non-acoustically resonant PA systems with broadband light sources and microphones are commonly used for the detection of gases such as carbon dioxide (CO_2_) [7,8], carbon monoxide (CO) [8,9] and methane (CH_4_) [7,8], which absorb infrared (IR) light strongly. Publications on photoacoustic gas sensors using a light-emitting diode (LED) as a light source and a microphone or a quartz tuning fork as an acoustic transducer can be found for CO_2_ detection [10,11] and methane detection in [12,13]. Laser-based systems can be found, for example, in [14] where a microphone is used as a transducer to detect CH_4_. Another example is an ethylene measurement device presented in [15], which uses a cantilever as a transducer. However, all the aforementioned publications on the state of the art describe systems that operate in the infrared wavelength range, so they are significantly different from the LED-based UV system described by us.

Since the emission power of UV-LEDs has increased significantly in recent years and the price has decreased at the same time, these light sources represent a very promising starting point to reduce the overall system costs of photoacoustic sensor systems. For this reason, we decided to combine commercially available UV-LEDs and MEMS-microphones with the aim to develop a sensitive, reliable, and price-reduced PA gas monitor with a high potential of commercialization.

We present a resonant photoacoustic (PA) combustion gas monitor that detects SO_2_ concentration variations of 1 ppm at gas flows in laboratory conditions [16]. The developed photoacoustic monitoring device detects SO_2_ in the UV region at 285 nm, using a commercially available UV LED and MEMS microphone [16]. Furthermore, the developed SO_2_ ship emission monitor was mounted to the funnel of a traveling container ship to conduct primary SO_2_ emission measurements.

## 2. The Photoacoustic Detection of SO_2_ Gas Emissions

### 2.1. Basics of the Photoacoustic SO_2_ Gas Detection

In the mid-infrared region, SO_2_ has several absorption bands in the range of the 7 to 9 µm wavelengths. Yin et al. presented in [17] a photoacoustic sensor system for the detection of SO_2_ in the ppb range, which uses a 7.41 µm external-cavity quantum cascade laser as light source. In this wavelength range, however, water also absorbs very strongly, which makes the detection of SO_2_ exiting an EGCS nearly impossible. In addition, light sources and detectors for the mid-infrared region are very expensive. For these reasons, we decided to detect SO_2_ in the ultraviolet wavelength range using the photoacoustic measurement principle.

For several years, various groups have been working on the development of UV-based SO_2_ measurement systems using lasers as the light source. However, to our knowledge, no LED-based photoacoustic measurement system has been described so far.

For example, in [18], Somesfalean et al. presented a laser-based method for sulphur dioxide spectroscopy in the UV region at a wavelength of 302 nm, which uses a photomultiplier tube as a detector. In [19], a SO_2_ photoacoustic sensor using a mW-level diode-pumped solid-state laser that emits at 303 nm is presented. Although these measurement systems are highly sensitive, they also require expensive and complicated optical systems with the necessary analysis setups, which makes a commercialization of these sensor systems considerably difficult. According to the SO_2_ emission limits set by the International Maritime Organization (IMO), a measurement range of 0–2000 ppm SO_2_ and a sensor detection limit of 2 ppm SO_2_ with an accuracy and precision of ±2 ppm within the SO_2_ concentration range 0–100 ppm and an accuracy and precision of ±6 ppm within the range of 100–2000 ppm SO_2_ would suffice. These are the key specifications during the development process of the novel and here presented SO_2_ sensor system.

The UV absorption spectrum of sulphur dioxide starts at the wavelength 390 nm with the very weak A-band, which is based on a spin-forbidden transition [20]. At higher energies a stronger absorption at the B-band of SO_2_ between the wavelengths 340 and 260 nm, often called the Clements band, is observed [21]. The B-band has a complex structure. At even higher energies, the C-band can be found with the strongest absorption of SO_2_ in the wavelength range from 180 to 240 nm. The C-band is based on dipole allowed transitions [22]. For the measuring system the absorption at the B-band was selected, specifically the range around 285 nm wavelength. The reasons for this are the availability of commercially available and affordable LEDs as a light source for that wavelength, low cross-sensitivities and that the energy of the radiation is not yet sufficient to dissociate most of the gas molecules. At 285 nm wavelength, there are significant cross-sensitivities to gaseous acetone, which is not relevant for marine applications, as well as minor cross-sensitivities to nitrous dioxide and ozone.

Regarding the photoacoustic measurement method, the Franck–Condon principle states, that the absorption of a photon is an instantaneous process, at which the nuclei are much heavier than electrons [23]. During light absorption, electrons can move, while the heavier atomic nuclei have no time to readjust themselves [23]. As a result, the atomic nuclei readjust after the absorption process, which is equivalent to generating vibrations [23]. According to this principle, the electronic transition in a SO_2_ molecule causes a charge redistribution in the molecule at a wavelength of 285 nm. This results in a change in Coulomb forces on the nuclei, which creates the changes in the vibrational state of the molecule [24].

**Figure 1:**
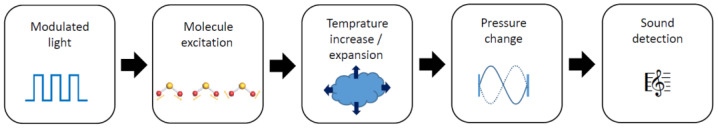
The photoacoustic detection principle of gases. Gas molecules absorb light at a specific wavelength, which excites the molecules to a higher energetic state. This results in a temperature increase and a consequent gas volume expansion, which is equivalent to a pressure change. A periodic modulation of the light source leads to a modulation frequency-dependent pressure change, which is equivalent to sound waves that can be detected by a microphone.

The PA signal is mainly generated by the colliding molecules, which cause a temperature increase (Figure 1) that results from the conversion of absorbed light energy to translational energy [5]. Assuming the ideal gas law, an increase in the gas temperature results an increase of the gas pressure. The modulation of a light source changes the gas temperature frequently, giving rise to a periodical pressure change (Figure 1) within a defined volume (typically a photoacoustic cell) [25]. The frequent pressure change within the photoacoustic cell can be considered as a sound signal that can be converted into an electric signal via an acoustic transducer [5].

### 2.2. The Photoacoustic Sensor System

**Figure 2:**
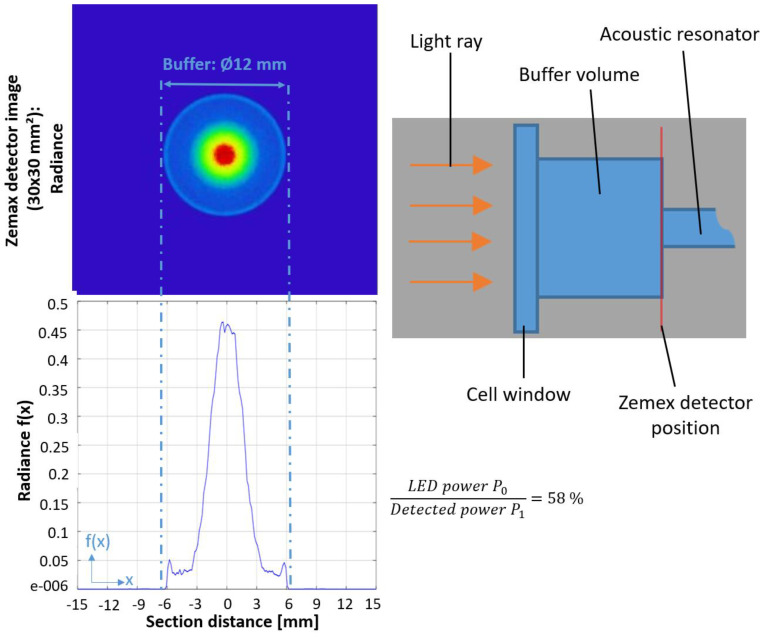
The ray-tracing simulation results with ZEMAX conducted at the entrance of the resonator (see simulation detector position in right schematic image). The focused beam diameter is clearly smaller than the buffer diameter (12 mm) and reaches a minimum diameter of around 2.8 mm before entering the photoacoustic resonator.

The design of the resonant photoacoustic cell essentially influences the sensitivity of the photoacoustic detector [5]. During a resonant operation, the exciting light source is modulated at a resonance frequency of the (typically cylindrically shaped) photoacoustic cell, which results in an amplification of the generated acoustic wave that corresponds to the photoacoustic signal [5]. Acoustic background signals, which are caused by absorptions of the resonator wall and the windows, limit the sensitivity and can be suppressed by large buffer volumes (Figure 2) on both sides of the cylindrical resonator [26].

The photoacoustic cell presented here (Figure 2) is made of stainless steel to withstand the corrosive gases and harsh conditions within a funnel bypass. The inner diameters of the cylindrical resonator and the two larger buffer volumes are set to 3 mm and 12 mm [16], while the lengths of the respective buffer volumes are set to 15 mm. To reduce the influence of lower frequented acoustic engine noise on the generated gas-dependent photoacoustic signal, the first longitudinal eigenmode of the resonator is set to 5 kHz, which is realized through a 30 mm-long resonator [16].

A high power AlGaN-based VPS174 UV LED (Nikkiso) with an output power up to 45 mW and an emitted light peak-wavelength of 285 nm is used for the photoacoustic SO_2_ sensor [27]. The large emission angle of the LED—about 130°—requires an additional optical lens to focus the light into the narrow resonator diameter [16,27]. Two UV light transmitting biconvex lenses and one planoconvex lens, made of quartz glass were used for the optical system. The two biconvex lenses have a diameter of 25.4 mm, with lens radii R_1_ and R_2_ of 31 mm and a focal length f of 35 mm. The planoconvex lens’ diameter is 12.7 mm with R_1_ being 9.2 mm and a focal length of 20 mm. Starting from the UV LED, the light first passes through the planoconvex lens and then the two biconvex lenses. The planoconvex lens focuses the LED light towards the first biconvex lens, which then collimates the emitted light, while the second biconvex lens focuses the UV light towards the entrance of the photoacoustic resonator. The exact positioning of the lenses was simulated via the ray-tracing and optical design software ZEMAX OpticsViewer. The simulation results state that the optimal distance between the planoconvex lens and the LED are to be set to 2 mm. The distance between the planoconvex and first biconvex lens is set to 19 mm. The distance between the two biconvex lenses is 11 mm, while the distance between the second biconvex lens and the photoacoustic resonator is set to 35 mm, which corresponds to the focal length f. The simulation results yield a beam diameter of 2.8 mm at the transition from the buffer volume to the resonator of the photoacoustic cell, which is shown in Figure 2.

**Figure 3:**
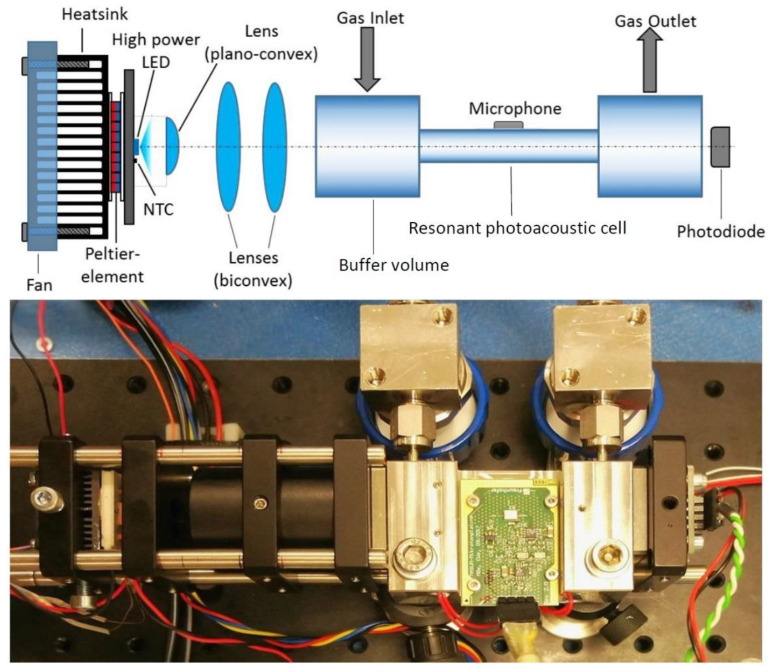
Setup of the developed resonant photoacoustic gas sensor. The upper part of the figure shows the sensor components in a schematic drawing. The sensor consists of the resonant photoacoustic cell with the MEMS microphone at the center of the resonator. The gas inlet and outlet are placed at the buffer volumes. The temperature of the high-power ultraviolet (UV) light-emitting diode (LED) is controlled via a thermoelectric cooler and a thermistor. The light beam is focused by optical lenses, which increases the sensitivity of the sensor system. An additional photodiode monitors the LED performance. The lower part of the figure is a photo of the corresponding developed sensor system [16].

The light source is electrically modulated with a square-wave function and a 50% duty cycle. To prevent the peak-wavelength of the high power LED from shifting due to heating of the LED, a temperature control (Figure 3) consisting of a thermistor (NTC), a thermoelectric cooler and a control unit stabilize the temperature of the light source [16]. To monitor the performance of the high-power LED, a photodiode is placed at the other end of the PA resonator, which is shown in Figure 3 [16].

After the inlet of the target gas, electromagnetic radiation emitted by the light source is absorbed and an acoustic signal is generated, which can be detected by a microphone. A MEMS microphone (ICS-40720; TDK) is placed at the centre of the resonator (Figure 3) to detect the maximum pressure change within the resonator. The microphone transduces the acoustic signal into an electric signal, which is further processed through a lock-in amplifier. The lock-in amplifier separates the measured photoacoustic signal from the unwanted noise [28]. Afterwards, the recorded raw measurement signal is analysed by the system electronics. The raw measurement data, as well as the analysis results, are transmitted via RS485 to a central computer, which collects various other sensor signals like ship speed, motor power and position beside the signals of the PA sensor system.

### 2.3. Sensor Signal Analysis

**Figure 4:**
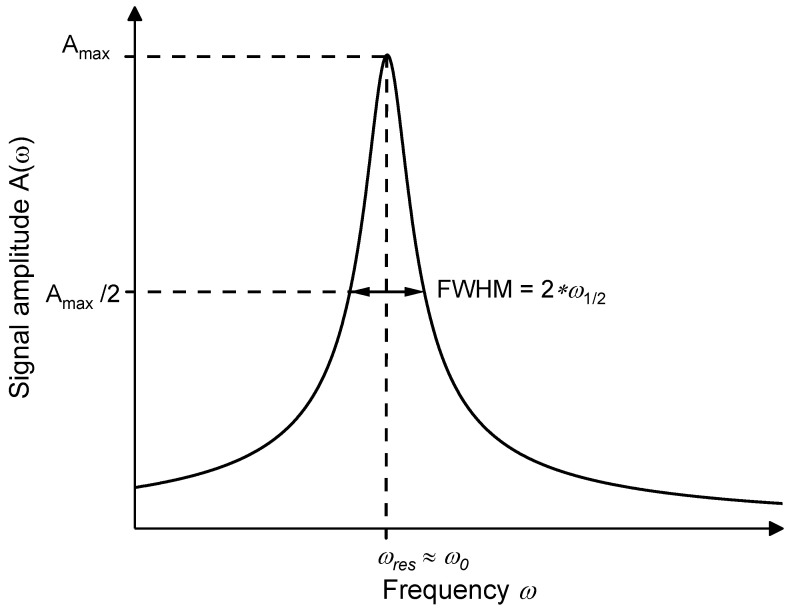
The resonance curve of a forced oscillation with full width at half maximum.

The external raw data analysis of the sensor signal is carried out using a Python script that identifies and determines the amplitude as well as the resonance frequency of every measured signal curve. The behavior of the photoacoustic resonance curve can be described as a forced oscillation (Figure 4), whose amplitude was described by Robert Wichard Pohl as the following Equation (1) [29]:

(1) $$A\left(\omega \right)=\frac{M_{0}}{\vartheta_{s}\cdot \sqrt{{{(\omega }_{0}^{2}{-\omega }^{2})}^{2}+\frac{\beta^{2}\omega^{2}}{\vartheta_{s}^{2}}}}$$

while the variables $M_{0},\;\beta$ and $\vartheta_{s}$ can be modified by the Equations (2)–(4) [29]:

(2) $$M_{0}{=A}_{\max}\cdot 2\cdot \omega_{0}\cdot \vartheta_{s}$$

and

(3) $$\beta =2\cdot \alpha \cdot \vartheta_{s}$$

as well as by describing α through the full width at half maximum

(4) Δω12=α·3.

Adding Equations (2)–(4) into Equation (1) simplifies the amplitude description formula A(ω) to Equation (5) [29]:

(5) $$A\left(\omega \right)=\frac{A_{\max}\cdot 2\cdot \alpha \cdot \omega_{0}}{\sqrt{{{(\omega }_{0}^{2}{-\omega }^{2})}^{2}+4\cdot \alpha^{2}\omega^{2}}}$$

The three remaining parameters—the amplitude $A_{max}$, the damping coefficient α and the frequency $\omega_{0}$—in Equation (5) can all be specified as starting values for the fit that describes the amplitude’s height, width and position within the frequency domain, which is essential for fusing the data analysis with the evaluation electronics.

**Figure 5:**
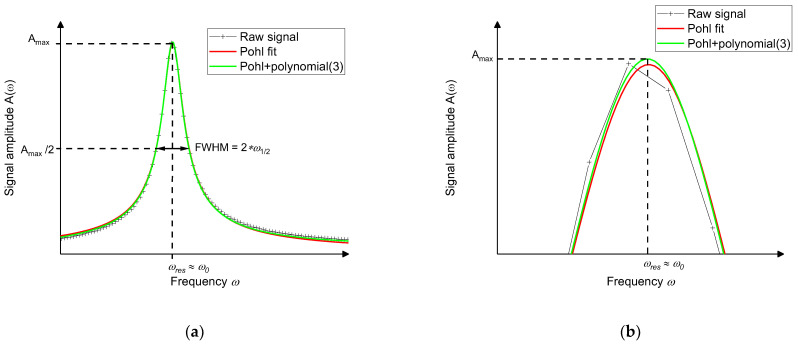
(**a**) The measured peak amplitude (black crosses) and the corresponding fit according to Equation (5) (red curve). The deviation of the red curve and the fitted curve occurs at the falling slope due to the short distance between the respective and the subsequent resonance peak resulting in an overlap of both peaks. By an additional third-degree polynomial, as introduced in Equation (6), the residuum of the fit improves by a factor of 4. The corresponding green curve has less deviation at the increasing and decreasing slopes compared to the red curve. (**b**) Image section of the peak maximum with a higher resolution: Signal noise could result in an incorrect determination of the amplitude peak height and resonance frequency. However, fitting a curve according to Equation (5) or (6) to the measured data, noise-related errors are reduced, which results in an accuracy increase of the sensor system. The differences between the two fitting procedures is less pronounced in this area but can still be seen.

Primary tests with the experimental data verified the correlation of the fit function in Equation (5) with the raw data signal curve of the resonant photoacoustic gas detector (Figure 5). Using the respective fit function minimized the point counts, which are required for a correct determination of the signal height (amplitude) and position within the frequency domain, to 20 points (Figure 5b). However, a perfect fitting with the experimental data is not granted at the increasing and falling slope, as can be seen in Figure 5a, due to the short distance between the respective resonance peak and the subsequent peak in the developed acoustic resonator, which results in an overlap of the falling and rising slopes of both resonance peaks. To improve the fitting precision, a third-degree polynomial was added to the theoretical formula from Equation (5). With this adjustment the residual $\chi^{2}$ was improved by a factor of 4.

(6) $$A\left(\omega \right)=\frac{A_{\max}\cdot 2\cdot \alpha \cdot \omega_{0}}{\sqrt{{{(\omega }_{0}^{2}{-\omega }^{2})}^{2}+4\cdot \alpha^{2}\omega^{2}}}{+a}_{1}\omega^{3}{+a}_{2}\omega^{2}{+a}_{3}{\omega +a}_{4}$$

Using this adjusted fitting equation, the precision of the fitting procedure and the measured data peak is adequate for the data analysis of all ongoing experiments.

## 3. SO_2_ Laboratory Measurements

**Figure 6:**
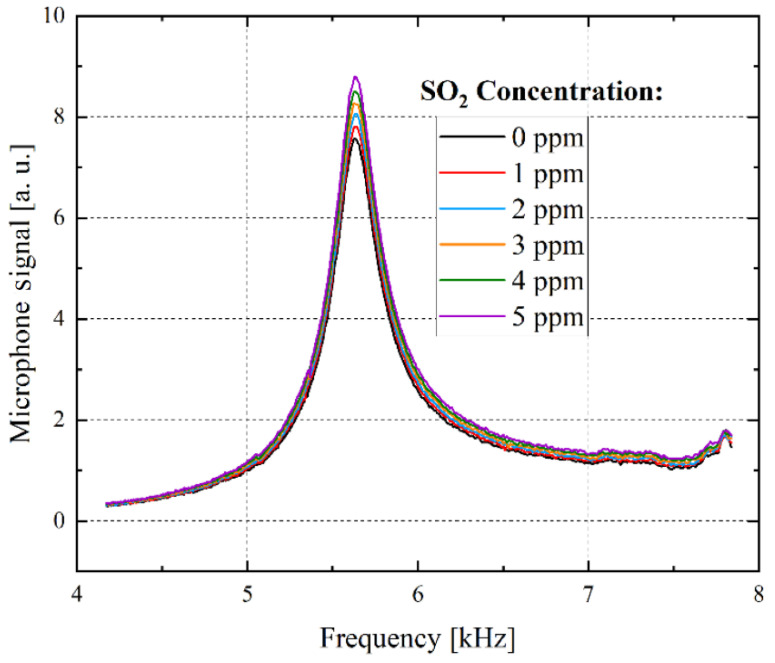
Sensor response during exposure to SO_2_. The SO_2_ concentration was varied from 0 to 5 ppm in 1 ppm steps. A resolution of one ppm is achieved. The microphone signal peak height increases with increasing SO_2_ concentrations [16].

SO_2_ lab measurements of the developed sensor system were carried out at a continuous gas flow of 200 mL/min, while the deployed UV-LED emits light at a central wavelength of 285 nm [16]. The SO_2_ concentration was varied in 1 ppm steps at a range between 0 and 5 ppm, as can be seen in Figure 6 [16]. A clear increase of the amplitude peak height with increasing SO_2_ gas concentration can be observed. A signal at 0 ppm SO_2_ can be detected (Figure 6), which can mainly be drawn back to the stainless steel wall absorptions of the photoacoustic measurement chamber that generate a detectable photoacoustic signal. The primary laboratory measurements shown in Figure 6 validate the required detection limit of 1 ppm of the developed photoacoustic SO_2_ sensor system.

**Figure 7:**
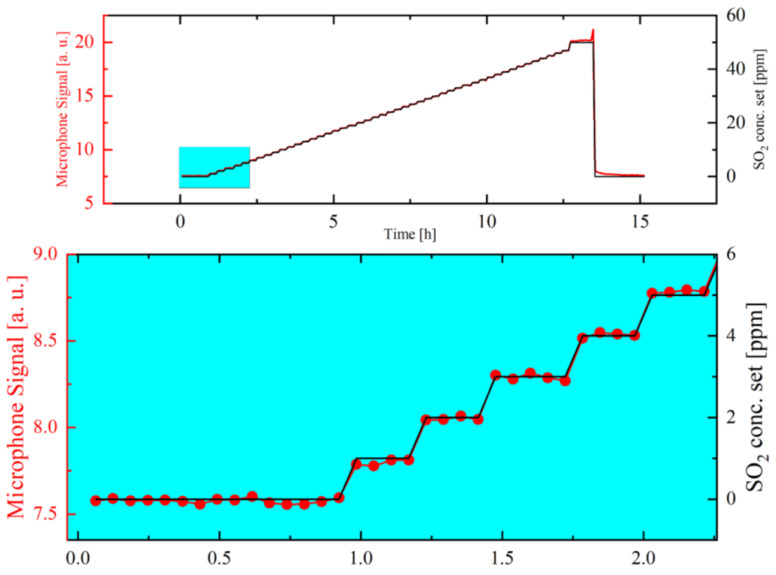
The microphone signal (red curve) in relation to the set SO_2_ concentration (black curve), which is varied between 0 and 50 ppm using pre-calibrated mass flow controllers. Upper graph: The detected microphone signal in relation to the set SO_2_ concentrations over a period of 15 h. Lower graph: zoom graph of the microphone signal at the first five concentration variations resembling the set SO_2_ concentrations 0 up to 5 ppm.

Furthermore, SO_2_ concentration variations starting at 0 ppm and going up to 50 ppm (1 ppm SO_2_ variation up to 47 ppm and the last step is a 3-ppm concentration variation) were performed at laboratory conditions using pre-calibrated mass flow controllers (MFC). The measurement shows a linear increase of the microphone signal with increasing set SO_2_ concentrations, as can be seen in Figure 7. Due to the direct relation between the microphone signal and the SO_2_ concentrations, which are set by the MFCs, the measured SO_2_ concentration can be estimated via the respective detected microphone signals. What can be drawn from Figure 7 additionally, is the background microphone signal at a set SO_2_ concentration of 0 ppm which increases by around 0.15 a.u. for every 1 ppm SO_2_ concentration increase. The background signal at 0 ppm SO_2_ is, as already explained, caused by absorption on the walls of the stainless-steel photoacoustic chamber. Laboratory tests examined the influence of external environmental conditions on the background signal. As the photoacoustic cell’s temperature is kept constant and adjusted via heating elements to a temperature of 50°C. In addition, the test gas temperature in the laboratory and during the field test were kept at a constant value as well, resulting in a stable behaviour of the detected background signal. No noteworthy signal change was observed during a 15-h laboratory measurement at 0 ppm SO_2_.

## 4. Field Test

**Figure 8:**
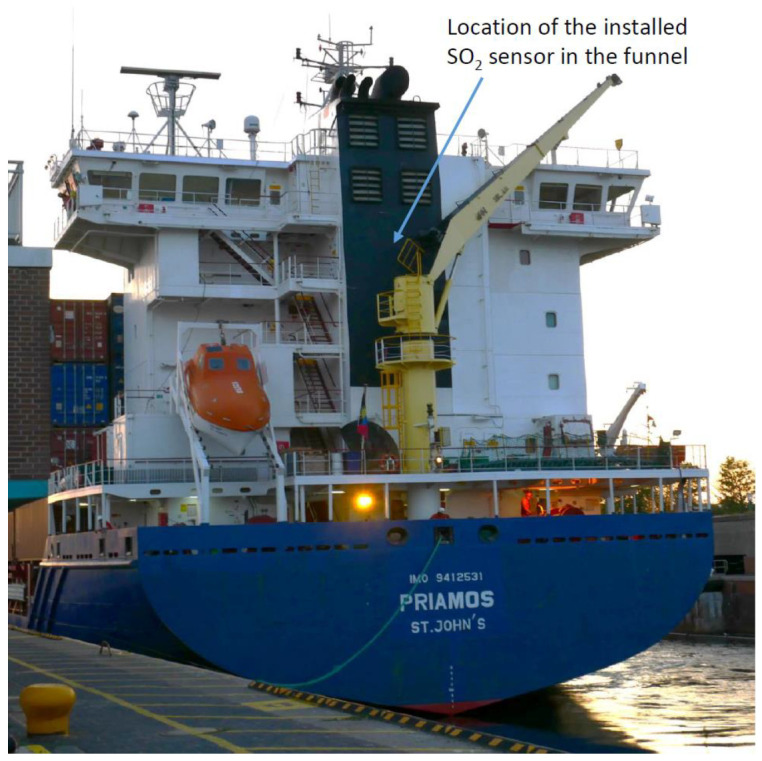
The container ship PRIAMOS with the approximate location of the installed and newly developed photoacoustic SO_2_ sensor.

The developed combustion monitoring system, which is shown in Figure 3, was integrated into the container ship PRIAMOS (Figure 8) that mainly operates within the North and Baltic Sea. PRIAMOS (IMO: 9412531) is a container ship (Cargo: Hazard A (Major)) that was built in 2011, which has an overall length of 157.71 m, is 23.54 m wide and shows a current draught of about 8.5 m. The container ship PRIAMOS sails under the flag of Antigua Barbuda and features a carrying capacity of 880 TEU (20 ft equivalent units). It only uses low sulphur fuel (maximum SO_2_ concentration about 109 ppm SO_2_) and subsequently does not require an additional EGCS to reach the allowed maximum emission values.

**Figure 9:**
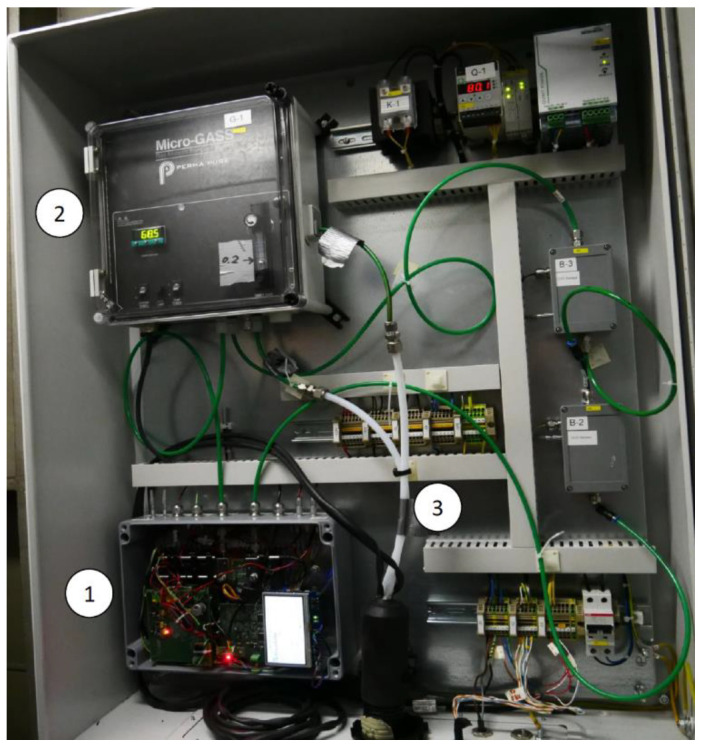
The internal components of the switch cabinet that were installed on the container ship PRIAMOS: (1) The developed photoacoustic SO_2_ sensor system. (2) The MicroGASS gas sampling system (Perma Pure, LCC) dries and cools the gas sample down. (3) Gas inlet and outlet tubes of the measured gas from and to the switch cabinet, which pass through the temperature-controlled pipe.

However, the emitted gas of a funnel with an EGCS shows typical temperatures around 50 °C, while the temperature of the emitted gas without an EGCS could reach 250 °C. Consequently, the emitted gas had to be cooled and dried—to prevent condensation—before analysing the gas in the developed monitoring system. A gas sampling system (MicroGASS, Perma Pure LLC) and a temperature-controlled preconditioning pipe (temperature controller: ST49, AGT-PSG GmbH and Co.KG) cooled the measuring gas probe to temperatures of about 60 °C. In addition, the pipe connected the ship funnel with the developed sensor system and a commercial exhaust gas analyser IMR 7500 (IMR Environmental Equipment, Inc.), which was installed as a reference monitoring device. The sensor system was integrated into a switch cabinet in combination with the gas sampling system (Figure 9), while the IMR 7500 reference system was installed separately on the container ship (but was connected to the sample pickup within the funnel).

**Figure 10:**
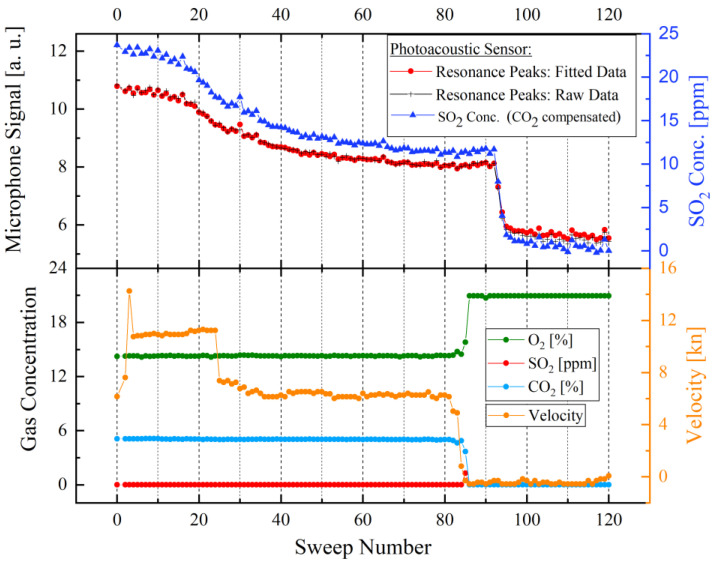
Measured raw data during container ship navigation. The upper diagram shows two different analysis methodologies of the measured data (red and black curve) by the developed SO_2_ sensor and the corresponding SO_2_ concentration in ppm after compensating the influence of CO_2_ on the detected signal (dark blue curve). The red curve in the upper diagram represents the data calculated through the instantaneous fitting process (via analysis electronics) of the measured data set, while the black diagram (with black crosses) corresponds to the subsequent manual analysis of the gathered data. The lower diagram shows the measured data by the IMR 7500 reference gas analyzer, which includes CO_2_, O_2_, SO_2_ concentrations and the measured velocity (kn ~ 0.514 m/s) of the container ship. At about sweep number 85 the engine is turned off, which results in the increase in the O_2_ concentration and a simultaneous reduction of the CO_2_ concentration. Subsequently, the measured SO_2_ concentration dependent microphone signal of the newly developed photoacoustic gas sensor reaches—with a delay at sweep number 90 caused by the additional gas volume of the tube connections—the lowest values.

After successfully installing the newly developed and the commercial gas analysers in the exhaust gas system and connecting them to the funnel, both sensor systems began acquiring the data of the test vessel. In Figure 10, the upper graph—the photoacoustic microphone signal of the developed SO_2_ sensor with the corresponding SO_2_ concentration; and lower graph—the measured gas concentrations measured by the IMR 7500; compare the measurements of the developed photoacoustic SO_2_ sensor to the measured values of the reference gas analyser [30]. All measured data in Figure 10 are plotted against the respective sweep number. The gas monitors were set to complete 120 sweeps per day [30]. The black curve in the upper graph in Figure 10 corresponds to the manually analysed raw data values of the measured peak maximum of every sweep, while the red curve complies with the values of the internal system analysis electronics [30]. Both curves are clearly matching, showing that the previously assumed fit function reliably represents the measured raw data. The dark blue curve in the upper graph represents the measured SO_2_ concentration in ppm after compensating the effect of CO_2_ on the detected signal.

Several important observations can be extracted from the collected data in Figure 10. The developed photoacoustic SO_2_ monitor successfully measures the fluctuations and changes of the SO_2_ content within the combustion emissions, which corresponds with the engine power of the container ship (represented by the orange curve (velocity) in Figure 10), as well as the abrupt falling CO_2_ and rising O_2_ concentrations (the light blue and green curves in Figure 10), which represent the switch-off of the engines. The time delay between the photoacoustic SO_2_ sensor system and the velocity, as well as the falling CO_2_ and rising O_2_ concentration values measured by the IMR 7500 occurs due to the additional gas volume (approximately 10 litres), which is formed by the connecting gas tubes and the required temperature-controlled pipe. Furthermore, the data in Figure 10 shows that the commercial gas analyser could not detect any concentration variations of the emitted SO_2_, which turned out to be caused by a defect in the IMR 7500 measurement system.

**Figure 11:**
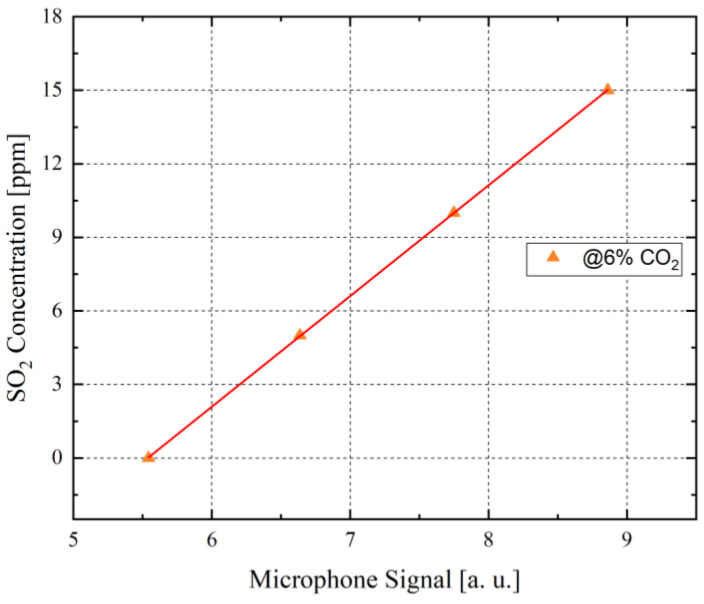
The microphone signal in relation to the set SO_2_ concentrations at a constant CO_2_ concentration of 6 vol.-%, using pre-calibrated mass flow controllers to conduct these measurements in the laboratory. The detected microphone signals and the set SO_2_ concentrations show a clear linear relation. An increase of the SO_2_ concentration increases the corresponding microphone signal. This graph is used as a reference to estimate the measured SO_2_ concentration from the detected microphone signal during the field test.

To translate the measured raw microphone signal values into a ppm concentration of the detected SO_2_, the values of the microphone signal are cross-referenced to a calibration curve, which was generated in laboratory conditions using pre-calibrated MFCs (Figure 11). Due to the significant influence of CO_2_ on the detected SO_2_ concentration in the ship funnel, the SO_2_ concentration calibration curve was generated at 6 vol.-% CO_2_, which corresponds with the detected CO_2_ concentration in the measured gas mixture within the funnel. Owing to the linearity of the calibration curve (Figure 11), the SO_2_ concentration in ppm can be estimated via the detected microphone signal. The determined ppm concentrations of the measured SO_2_ showed that the detected SO_2_ concentrations varied between 0 and 23 ppm, as can be seen in Figure 10. At the beginning of the measurements, the emitted SO_2_ concentration is at about 23 ppm, after which the container ship lowers its velocity and whereupon the measured SO_2_ concentration falls to about 11 ppm. At approximately sweep number 90 (Figure 10), the emitted SO_2_ concentration value falls to about 0 ppm, which represents the complete standstill of the container ship after reaching parking position at the targeted harbour. All presented SO_2_ concentration values are in accordance with the expected SO_2_ concentration range between 22 and 36 ppm SO_2_ that container ships which use low sulphur fuel must emit by law.

## 5. Conclusions and Outlook

The demand in reliable combustion emission-monitoring systems highly increased since the global restrictions of the container ship SO_2_ emissions came into force. Therefore, a photoacoustic based SO_2_ gas detector has been developed and was investigated. The SO_2_ gas monitor is based on the resonant photoacoustic detection, using commercially available MEMS microphones and UV LEDs.

Primary laboratory measurement results show the successful realization of a SO_2_ sensor system with a detection limit of 1 ppm. We are thus in the order of magnitude of the elaborate and very complex classic NDUV gas analyser MARSIC300 offered by SICK. Furthermore, the implementation of the SO_2_ monitoring device on an operating container ship and measuring the emitted SO_2_ concentration fluctuations in the combustion gas was successfully completed. Comparing the measured SO_2_ dependent microphone signal with the measured CO_2_ and O_2_ concentrations, as well as with the container ship velocity, showed an expected time delay, which is caused by the additional gas volume of the tube connections. In addition, the measurements yielded that the instantaneous analysis via fitting of the forced oscillation equation by Pohl with an additional mathematical adjustment to the measured raw data is a suitable analysis methodology for the sensor system realization.

After measuring an acoustic background signal at 0 ppm SO_2_ (Figure 5), further research is planned to reduce this unwanted effect. First tests were performed and showed a significant reduction of the photoacoustic background signal at 0 ppm SO_2_ when the dimensions of the resonator and buffer volume are increased. As a result, less light falls on the wall of the resonator chamber, which decreases wall absorption considerably, that subsequently reduces the background signal. Additionally, an optimization of the optical lens setup is planned to reduce the impingement of the UV light on inner walls of the acoustic resonator. In parallel, new UV-LEDs with integrated lenses are planned to be evaluated, to reduce the overall costs of the current optical system.

The demonstrator developed shows the potential for a future low-cost SO_2_ detector to meet the necessary requirements and specifications for monitoring the ship emissions. Further investigations regarding long-term stability and detection limits <1 ppm SO_2_ are currently underway. In addition, the influence of other gases contained in ship exhausts on the detected photoacoustic SO_2_ signal is being investigated.

## Data Availability

Data sets are available at http://dx.doi.org/10.24406/fordatis/129 (accessed date: 29 June 2021).
